# FASTAFS: file system virtualisation of random access compressed FASTA files

**DOI:** 10.1186/s12859-021-04455-3

**Published:** 2021-11-01

**Authors:** Youri Hoogstrate, Guido W. Jenster, Harmen J. G. van de Werken

**Affiliations:** 1 Department of Neurology, Erasmus University Medical Center, Dr. Molewaterplein 40, 3015 GD Rotterdam, The Netherlands; 2 Department of Urology, Erasmus MC Cancer Institute, University Medical Center, Rotterdam, The Netherlands; 3 Cancer Computational Biology Center, Erasmus MC Cancer Institute, University Medical Center, Rotterdam, The Netherlands; 4 Department of Immunology, Erasmus MC Cancer Institute, University Medical Center, Rotterdam, The Netherlands

**Keywords:** FASTA, FASTAFS, Integrity, FUSE, Zstd, Metadata, Random access, Virtualisation

## Abstract

**Background:**

The FASTA file format, used to store polymeric sequence data, has become a bioinformatics file standard used for decades. The relatively large files require additional files, beyond the scope of the original format, to identify sequences and to provide random access. Multiple compressors have been developed to archive FASTA files back and forth, but these lack direct access to targeted content or metadata of the archive. Moreover, these solutions are not directly backwards compatible to FASTA files, resulting in limited software integration.

**Results:**

We designed a linux based toolkit that virtualises the content of DNA, RNA and protein FASTA archives into the filesystem by using filesystem in userspace. This guarantees in-sync virtualised metadata files and offers fast random-access decompression using bit encodings plus Zstandard (zstd). The toolkit, FASTAFS, can track all its system-wide running instances, allows file integrity verification and can provide, instantly, scriptable access to sequence files and is easy to use and deploy. The file compression ratios were comparable but not superior to other state of the art archival tools, despite the innovative random access feature implemented in FASTAFS.

**Conclusions:**

FASTAFS is a user-friendly and easy to deploy backwards compatible generic purpose solution to store and access compressed FASTA files, since it offers file system access to FASTA files as well as in-sync metadata files through file virtualisation. Using virtual filesystems as in-between layer offers format conversion without the need to rewrite code into different programming languages while preserving compatibility.

**Supplementary Information:**

The online version contains supplementary material available at 10.1186/s12859-021-04455-3.

## Background

FASTA is a file format used for storing nucleotide and amino acid polymeric sequences and is compatible with a high variety of bioinformatics software. It is used as database for ribosomal RNA sequences but also for eukaryotic reference genomes and protein databases, that can be several gigabytes in size. In contrast to for example the GenBank format, it offers limited support for metadata. Corresponding supplementary (*fai*-)index files are used to achieve random access by providing the sequence length, padding corrected file positions and padding and line length. This is static information that is embedded in the FASTA file, which is extracted after generating the FASTA file.

Scientific demand for reproducibility and interoperability of both software applications and data is growing strongly and as a result unique identification and data integrity play a critical role. For instance, in the CRAM data format Next Generation Sequencing (NGS) alignments are compressed relative to a reference sequence. In this format, these reference sequences are addressed using their unique identifier for interoperability. With this identifier, the corresponding sequence can be obtained directly using the online European Nucleotide Archive (ENA) service^1^, preserving the intrinsic link between the data file and the reference sequences. Because real-time computation of identifiers can be computationally expensive, they are stored in supplementary dictionary files (*.dict). *Dict-*files are, like *fai-*index files, beyond the scope of the original file format and have to be generated and maintained after obtaining the FASTA file.

Software applications make use of FASTA files as input in two different manners:

- First, a tool reads a FASTA file in a ${\textit{streaming}}$ *manner*: sequentially and in one-direction, starting with the first character in the file. For example, short-read alignment algorithms but also motif-scanners that iteratively search for a given motif [1] across a sequence, read a FASTA file sequentially into the memory before building an index [2, 3]. Similarly, Single-Nucleotide Polymorphism (SNP) detectors may iterate sequentially over a FASTA file [4].
- Second, a tool reads a FASTA file in a ${\textit{random-access}}$ *manner* by starting at an arbitrary location in the file and having the possibility to make jumps through the file, forwards but also backwards. The precise file coordinates are typically calculated using the *fai-*index file. For example, a request to a genomic region within a genome browser is such a random-access request, since a next query can be expected at any genomic location. If underlying FASTA file access does not support jumping through a file, it is necessary to copy a file entirely into memory. This procedure is resource intensive and can slow a process significantly. Bioinformatics tools that rely on random-access in FASTA files are for i.e. JBrowse [5], samtools mpileup for VarScan2 [6]. But also tools for quick file operations such as SeqKit [7], GATK [8] and Picard [9] rely on random-access, of which the latter two require *dict-*files as well.

### Compression

The simplicity of the FASTA format makes it highly convenient to work with. The trade-off is the requirement of the additional *fai-*index and *dict-*files, as well as having a relatively large file size. The large file size issue has been tackled by various compression methods [10–12]. Although modern compressors achieve high compression ratios, most bioinformatics applications that require FASTA files are only rarely compatible with compressed equivalents. The only exception is occasional compatibility with gzipped FASTA, a generic purpose compressor which is limited to streaming use-cases.

Sequence compression algorithms create a compressed file (archive) yielding the compressed content. To use the original data, the archive needs to be fully decompressed into a temporary FASTA file again, unless the decompression algorithm also provides an Application Programming Interface (API) in the desired programming language. For instance, short read compressor DNA Sequence Reads Compressor 2 (DSRC2) [13] provides an API in C, C++ and Python.

The index algorithm of RNA read aligner STAR [3] can be provided with the path to any decompression binary as argument and thus offers a generic solution to provide on-demand de-compression. However, implementing a similar solution in other applications would only work for applications with streaming instead of random access to FASTA files. An analogues workaround to avoid file duplication is to make use of (named) pipes [10]. A pipe is a virtual, one-directional, data stream, that stays in idle as long as no further data requests come in. This could be the output of a decompressor. This is resource efficient as data access is chunked, but is not a generic solution as it does not offer random access. Access to FASTA archives in a random-access use case requires an available compression API that supports random access explicitly. If these conditions are not met, the primary goal of compression is then in practice lost.

Currently available bioinformatics applications that make use of FASTA files in a random-access setting mostly support only FASTA files and no compressed equivalents. Therefore, it is in practice necessary to keep a flat copy of a FASTA file with the corresponding the *fai-*index file. For systems limited to applications with streaming access to FASTA files, a decompression binary in combination with (named) pipes is an ideal way to use FASTA archives, although it requires management of metadata files. Instead of (de)compressing through the classical back and forths file compression binaries, we can also make use of file virtualisation. In this context, file virtualization functions as layer between a compressed archive and the virtually mounted FASTA plus metadata files, which offers multiple advantages over classical (de-)compression binaries

- Virtual files and their system calls are identical to flat file system calls. For command line tools that are only compatible with FASTA files, such as samtools view -T, this preserves backwards compatibility, also for random access use-cases.
- There is no need to use additional disk space for temporary decompression and no need to read entire FASTA files into memory.
- For random access requests, computational resources are only spent on decompressing the region of interest.
- Implementations of compression and decompression in other programming languages or within other software applications are not needed, as it is backwards compatible with flat FASTA files.
- The archive is guaranteed to provide *dict-* and *fai-*index files that are in sync with their FASTA file of origin. This makes additional management of these metadata files unnecessary.

Making use of virtualization as layer between archive and decompressed content is a generic purpose solution since it provides file access to both streaming and random access. Here, we propose FASTAFS, a file archival format and toolkit that allows file integrity verification and provides unique sequence identifiers by using file virtualization. In addition, it virtualises FASTA and guaranteed in-sync *dict-* and *fai-*index files, from Zstd compressed 2-, 4- or 5-bit encodings.

## Implementation

**Fig. 1:**
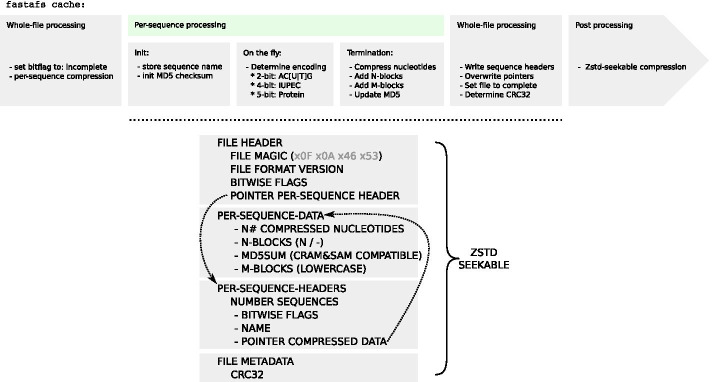
Overview of the FASTAFS file format specification. (top) A flowchart of the fastafs cache procedure. (bottom) The layout of the FASTAFS format consists of four blocks, starting with the file header, followed by the per-sequence data, the per-sequence header data and a metadata block. The file header has a file pointer to the per-sequence header block, where each sequence has a file pointer to its data. The file ends with a metadata block, currently supporting a CRC32 checksum. The raw FASTAFS file is subsequently compressed with zstd-seekable. The full specification is available on the website: https://github.com/yhoogstrate/fastafs/blob/master/doc/FASTAFS-FORMAT-SPECIFICATION.md

FASTA File System (FASTAFS) file format consists of four blocks: (1) File Header (2) Per-Sequence-Data (3) Per-Sequence-Header and (4) File Metadata, to efficiently store sequence and metadata (Fig. 1). During conversion, the metadata flag sets the archives status to incomplete. Each block of compressed sequence data is followed by the CRAM format and BAM specification compatible MD5 checksum [14, 15]. In the last phase of file conversion, file pointers are put in place and the metadata flag is updated to mark the archives conversion status to complete. The file ends with the CRC32 checksum used for whole file integrity verification.

Sequence compressor Nucleotide Archival Format (NAF) [10] compresses sequence data first with a 4-bit encoding followed by generic compressor Zstandard (zstd), but lacks random access. Given that NAF achieves high compression ratios [10], FASTAFS was designed in a similar fashion. It first compresses sequence data to a lower bit encoding, followed by the random-access implementation of zstd (zstd-seekable). When a sequence consists of AC[T|U]GN, it is stored in a 2-bit encoding, when it follows the IUPAC DNA/RNA dictionary, it is stored in a 4-bit encoding and when it is a protein sequence it is stored in a 5-bit encoding (Fig. 1).

### Toolkit

The Linux based FATSTAFS toolkit is a single executable (fastafs) with different subcommands. The package comes also with an executable ‘mount.fastafs’ to mount through the /etc/fstab table.

**Cache: ** FASTA files can be converted to a FASTFS archive using the ‘fastafs cache’ subcommand (Fig. 1), which adds a reference to the FASTAFS file into a config-file (Additional file 1: Fig. S1A).

**Mount: ** The ‘fastafs mount’ subcommand is used to mount a FASTAFS archive to a directory (mount point) to virtualise the FASTA, *fai-*index, *dict-* and UCSC TwoBit files (Additional file 1: Fig. S1A). All files are mounted read-only which guarantees persistency with the identifiers. Mount points can be configured in /etc/fstab which requires using the binary ‘mount.fastafs’ instead of the binary ‘fastafs’. These entries can be configured to automatically mount during boot. Upon a file request, the kernel requests, through the Filesystem in Userspace (FUSE), the FASTAFS toolkit to provide either file attributes such a timestamps, size or permissions, or to copy real-time decompressed file content into a buffer.

In addition, FASTAFS provides filesystem access to query partial sequences using a subsequence identifier as filename in the ‘seq’ subdirectory. For example, the file <mountpoint>/seq/chr1:10-20 contains only the 10th up to the 20th nucleotide of chr1, without additional characters such as newlines or spaces. Subsequently, requesting the file size of <mount point>/seq/chr1 will provide its size in nucleotides. Indeed, these additional features do not solve backwards compatibility issues, but do provide virtualised random access by functioning as programming language independent API implemented at filesystem level.

**List: ** The ‘fastafs list’ command gives an overview of the FASTAFS archives, their alias, number of sequences, format, compression ratio and all active mount points (Additional file 1: Fig. S1A).

**View: ** Besides mounting, the FASTA contents can be decompressed to *stdout* using ‘fastafs view’, of which the padding can be set to a desired value and masking can be virtually disabled. If the archive contains only DNA sequences, the contents can also be exported to the UCSC TwoBit format (Additional file 1: Fig. S1B).

**Info: ** The ‘fastafs info’ subcommand gives information about the file layout, sequence size, the per-sequence MD5 checksum and used compression type. This subcommand can also be used to query European Nucleotide Archive (ENA) [16] whether the existence of a sequence MD5 checksum can be verified (Additional file 1: Fig. S1C).

**Check: ** The ‘fastafs check’ command checks the file integrity using a CRC32 checksum. Integrity of compressed sequence data blocks can be checked separately using their MD5 checksums with the ‘--﻿md5’ argument (Additional file 1: Fig. S1D).

**ps: ** A list of active FASTAFS mount points and their processes is provided by the ‘fastafs ps’ subcommand. The mount point has an extended file attribute (*xattr*) named ‘*FASTAFS-file*’ that returns the path to the mounted FASTAFS archive. When a FASTAFS file is mounted to multiple mount points, they are each listed as separate entry with the corresponding system process id (Additional file 1: Fig. S1E).

## Results

FASTAFS format specification, toolkit and GPL-2.0 licensed C$++$ code are available at: https://github.com/yhoogstrate/fastafs

**Fig. 2:**
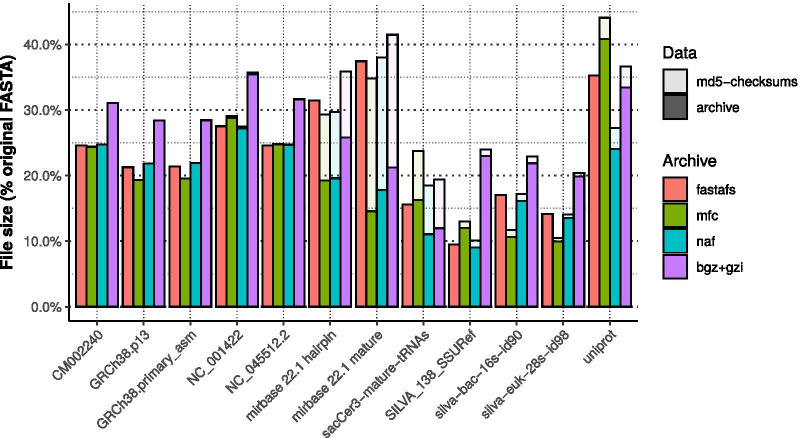
Overview of different archive files sizes. Comparison of compression ratios of a diverse set of FASTA files compressed with bgzip, MFCompress, NAF and FASTAFS. The bar height represents the percentage of the archives file size compared with the original FASTA files size. The translucent bars on top of the coloured bars represent the corrected file size needed to store 16 additional bytes per-sequence reserved for storing MD5 checksums. We used genome references from fungi (CM002240), human with and without alternate loci (GRCh38.p13 and GRCh38.primary_asm), DNA (Coliphage phi-X174: NC_001422) and RNA viruses (SARS-CoV-2: NC_045512.2), databases with small RNAs (miRbase and tRNAs), Silva rRNA databases [17] and uniprot [18] for protein sequences

**Fig. 3:**
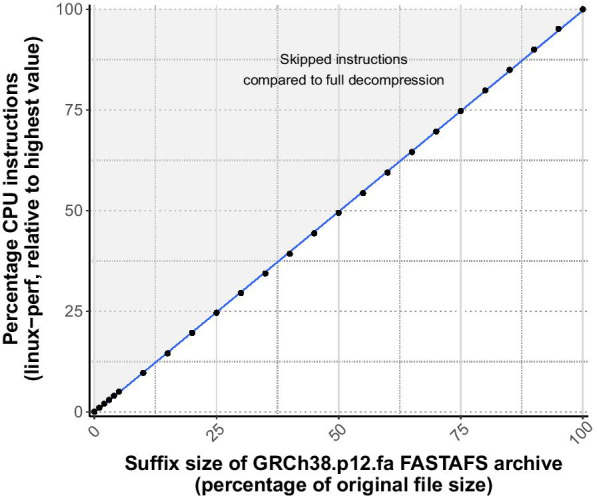
CPU consumption reading suffixes of the FASTA file. The x-axis shows the size of a requested suffix (tail-c) of GRCh38.p12.fasta virtualised with FASTAFS, piped through to /dev/null. The y-axis shows the number of CPU instructions by fastafs mount to virtualise the suffix of GRCh38.p12.fasta, as termined with perf stat. The number of CPU instructions needed to read the FASTA suffix through FASTAFS scales linearly and proportionally with the suffix size

We compared the compression ratios of NAF, bgzip and MFCompress with FASTAFS (Fig. 2). We then assessed the consumption of the relative number of CPU instructions needed to access the end, thus the suffix, of a virtualised FASTA file. This showed that the number of CPU instructions scaled linearly and proportionally to the suffix size (Fig. 3).

## Discussion

FASTAFS compression ratios for FASTA files with relatively few sequences (human reference genome: GRCh38, SARS-CoV-2 genome primary assembly (RNA): NC_045512.2, Coliphage phi-X174, complete genome NC_001422, fungus Neurospora crassa genome reference: CM002240) were comparable to the compression ratios of NAF and MFCompress but were overall not superior. For sequences with relatively high numbers of sequences (miRNA, tRNA or protein databases), compression ratios of FASTAFS files were typically smaller than the other compressors, in particular for miRbase [19]. These files are composed of small sequences which result in a substantial contribution of the sequence names and MD5 checksums to the total archive file size. When the size of the archives is corrected with the space needed to store the MD5 checksums (16 bytes per entry), the FASTAFS compression ratios are again comparable to those of MFCompress and NAF. Except for protein sequence compression, the most commonly used FASTA compression method, gzip, has consistently lower compression ratios than all other compressors.

Obtaining both the FASTA followed by the *fai-*index starting from a NAF archive took more CPU instructions than virtual access through FASTAFS (Additional file 1: Fig. S2). Moreover, classical decompression through a single conversion binary is not linearly scalable and requires the whole decompressed file to be written to disk. Although this is not the use-case for which these tools were developed and therefore maybe not represent the best comparison, these are features that provide high usability.

Ideally, new bioinformatics analysis projects are started with a new folder that is under version control. This will allow the researcher to integrate FASTAFS with workflow management systems such as Snakemake [20] or Nextflow [21] as well as software dependencies by including dependency management configurations. Ultimately, this makes a project portable as it allows users to distribute projects over multiple locations, share it with other researchers and roll back to previous versions. Currently, version control for plain FASTA files is inconvenient and redundancy across multiple projects will occur quickly. However, by integrating FASTAFS mount points and scripts into a workflow management system FASTA files can be integrated intuitively into a projects’ version control.

FASTAFS archives are currently compressed with a 2-bit, 4-bit or 5-bit encoding, followed by zstd-seekable, resulting in comparable compression ratios to other known compressors. Indeed, as may be expected from compression supporting random access which also need to store their index indexes, FASTAFS compression ratios were not superior in all cases examined. However, differences in compression ratios were consistently close to other dedicated compressors and always outperformed commonly used gzip, while offering all the FASTAFS advantages has.

FASTFS currently works with per-file aliases and CRAM compatible per-sequence identifiers. It would be more convenient to integrate FASTA files into workflow managers by using persistent per-file identifiers combined with a mechanism for decentralised synchronization of archives. As such additional features would be helpful, defining a system for per-file identifiers and development of decentralised file synchronization prompts future work. Overall, FASTFS is a modern and highly elegant software solution for a user-friendly and easy to deploy generic purpose solution to store and access to compressed FASTA files.

Multiple system processes can, in parallel, request data from the same FASTSAFS mount point. The current implementation is single-threaded per filehandle, but thread-safe. Thread safety is required because the Linux kernel is not guaranteed to request data sequentially during sequential reads. These non-sequential data requests will make optimization of FASTAFS for multi-threading more complicated, but will be a logical and important next step.

## Conclusions

The FASTA file format is used to store biological polymeric sequence data in an easy-to-use format that has become a file standard in bioinformatics. Static information is embedded within each file, but needs to be extracted and stored in additional files to complement the FASTA file. Previous methods have specifically focused on the most efficient compression possible, but not on backwards compatibility, interoperability, random access and inclusion of (static) metadata. This is the most probable explanation why gzip, a generic purpose compression method that is suboptimal for this data type, is the most common integrated archive type in bioinformatics applications that use FASTA as input.

**Fig. 4:**
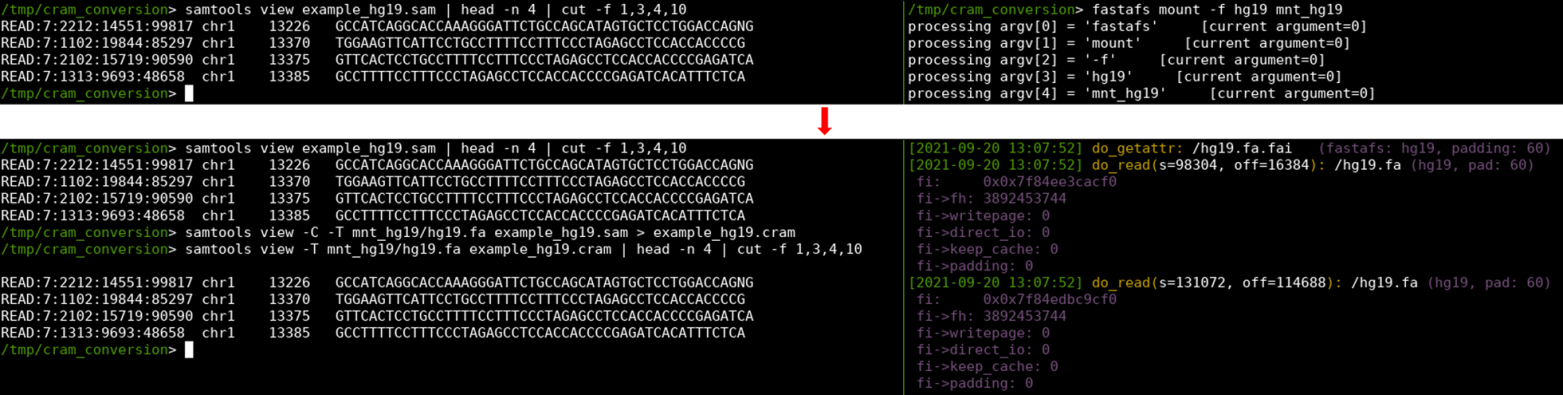
Screenshot showing compatibility with ’samtools view’ for CRAM files. The right panel shows an active fastafs mount process with debug messages enabled, mounting the hg19 FASTAFS archive to ./mnt_hg19. The left panel shows three samtools operations: Command 1 shows the original SAM-file. Command 2 runs the samtools conversion from SAM to CRAM, requiring the mounted FASTA file by the mount process (right panel). Command 3 shows the contents of the CRAM file. The CRAM file lacks actual sequence data, which samtools needs to extract from the FASTA file mounted in the right panel. The FASTAFS mount process (right) shows that samtools has requested access to both ./mnt_hg19/hg19.fa and ./mnt_hg19/hg19.fa.fai

To address this, we developed FASTAFS, a toolkit to virtualise FASTA archives along with their metadata files into the file system. The implementation makes use of the zstd-seekable compression library, which makes random access to the virtual FASTA files possible. FASTAFS comes with a feature rich toolkit that can manage the archives, their locations, their file integrity and provides file access in a backwards compatible manner to regular FASTA file access. This allows the archives to be used with existing software, such as samtools (Fig. 4), without the need for adaptation for compatibility or without requiring the presence of APIs or without requiring to fully decompress data to disk before accessing it. FASTAFS is a modern, versatile and elegant solution for storage of sequence data that is therefore easy to use, and can optionally be extended with other related file formats.

## Supplementary Information

**Additional file 1**. Supplementary Figures.

## Data Availability

**FASTAFS:** **Project name:** fastafs; **Project home page:**https://github.com/yhoogstrate/fastafs; **Operating system(s):** POSIX compliant; **Programming language:** C++-14; **Other requirements:** cmake, C++-14 compatible compiler, libzstd, libopenssl, libcrypto, zlib and libboost (for unit testing); **License:** GNU GPL-2.0; **Any restrictions to use by non-academics:** terms stated in GNU GPL-2.0

## Abbreviations

API: Application programming interface
DSRC2: DNA sequence reads compressor 2
ENA: European nucleotide archive
FUSE: Filesystem in userspace
NAF: Nucleotide archival format
NGS: Next generation sequencing
SNP: Single-nucleotide polymorphism
Zstd: Zstandard

## Footnotes

1 https://www.ebi.ac.uk/ena/cram/swagger-ui.html.

## Declarations

### Sequences

All sequences used were obtained from free public resources: **CM002240:** https://www.ebi.ac.uk/ena/browser/view/CM002240; **hg19.fa:** http://hgdownload.cse.ucsc.edu/goldenpath/hg19/bigZips/hg19.fa.gz; **GRCh38.p12:** http://ftp.ebi.ac.uk/pub/databases/gencode/Gencode_human/release_28/GRCh38.p12.genome.fa.gz; **GRCh38.p13:** http://ftp.ebi.ac.uk/pub/databases/gencode/Gencode_human/release_38/GRCh38.p13.genome.fa.gz; **GRCh38.primary**_**asm:** http://ftp.ebi.ac.uk/pub/databases/gencode/Gencode_human/release_38/GRCh38.primary_assembly.genome.fa.gz; **NC**_**001422:** ftp://ftp.ncbi.nlm.nih.gov/genomes/Viruses/enterobacteria_phage_phix174_sensu_lato_uid14015/NC_001422.fna; **NC**_**045512.2:** https://github.com/PoonLab/covizu/blob/master/covizu/data/NC_045512.fa; **miRBase 22.1 hairpin:** ftp://mirbase.org/pub/mirbase/CURRENT/hairpin.fa.gz; **sacCer3-mature-tRNAs:** http://gtrnadb.ucsc.edu/genomes/eukaryota/Scere3/sacCer3-mature-tRNAs.fa; **SILVA**_**138**_**SSURef:** https://www.arb-silva.de/no_cache/download/archive/release_138/Exports/; **silva-bac-16s-1d90:** https://raw.githubusercontent.com/biocore/sortmerna/v4.2.0/data/rRNA_databases/silva-bac-16s-id90.fasta; **silva-euk-28s-id98:** https://raw.githubusercontent.com/biocore/sortmerna/v4.2.0/data/rRNA_databases/silva-euk-28s-id98.fasta; **uniprot**_**sprot:** https://ftp.uniprot.org/pub/databases/uniprot/current_release/knowledgebase/complete/uniprot_sprot.fasta.gz;

## References

[1] Heinz S, Benner C, Spann N, Bertolino E, Lin YC, Laslo P, Cheng JX, Murre C, Singh H, Glass CK (2010). Simple combinations of lineage-determining transcription factors prime cis-regulatory elements required for macrophage and b cell identities. Mol. Cell.

[2] Kopylova E, Noé L, Touzet H (2012). SortMeRNA: fast and accurate filtering of ribosomal RNAs in metatranscriptomic data. Bioinformatics.

[3] Dobin A, Davis CA, Schlesinger F, Drenkow J, Zaleski C, Jha S, Batut P, Chaisson M, Gingeras TR (2012). STAR: ultrafast universal RNA-seq aligner. Bioinformatics.

[4] Liao Y, Smyth GK, Shi W (2013). The Subread aligner: fast, accurate and scalable read mapping by seed-and-vote. Nucleic Acids Res.

[5] Buels R, Yao E, Diesh CM, Hayes RD, Munoz-Torres M, Helt G, Goodstein DM, Elsik CG, Lewis SE, Stein L, Holmes IH (2016). Jbrowse: a dynamic web platform for genome visualization and analysis. Genome Biol.

[6] Koboldt DC, Zhang Q, Larson DE, Shen D, McLellan MD, Lin L, Miller CA, Mardis ER, Ding L, Wilson RK (2012). Varscan 2: somatic mutation and copy number alteration discovery in cancer by exome sequencing. Genome Res.

[7] Shen W, Le S, Li Y, Hu F (2016). Seqkit: a cross-platform and ultrafast toolkit for fasta/q file manipulation. PloS One.

[8] McKenna A, Hanna M, Banks E, Sivachenko A, Cibulskis K, Kernytsky A, Garimella K, Altshuler D, Gabriel S, Daly M, DePristo MA (2010). The genome analysis toolkit: a mapreduce framework for analyzing next-generation dna sequencing data. Genome Res.

[9] Picard toolkit. Broad Institute (2019). https://broadinstitute.github.io/picard

[10] Kryukov K, Ueda MT, Nakagawa S, Imanishi T (2019). Nucleotide archival format (NAF) enables efficient lossless reference-free compression of DNA sequences. Bioinformatics.

[11] Pinho AJ, Pratas D (2013). MFCompress: a compression tool for FASTA and multi-FASTA data. Bioinformatics.

[12] Rajarajeswari P, Apparao A (2011). Dnabit compress-genome compression algorithm. Bioinformation.

[13] Roguski L, Deorowicz S (2014). DSRC 2-industry-oriented compression of FASTQ files. Bioinformatics.

[14] Samtools Organisation: CRAM format specification (version 3.0: 2fcaab6). https://samtools.github.io/hts-specs/CRAMv3.pdf (2019)

[15] The SAM/BAM format specification working group: sequence alignment/map format specification (version 1.6: f2a6b99). 2019. https://samtools.github.io/hts-specs/SAMv1.pdf.

[16] European Bioinformatics Institute: CRAM reference registry. https://www.ebi.ac.uk/ena/cram (2019)

[17] Quast C, Pruesse E, Yilmaz P, Gerken J, Schweer T, Yarza P, Peplies J, Glöckner FO (2013). The SILVA ribosomal RNA gene database project: improved data processing and web-based tools. Nucleic Acids Res.

[18] Apweiler R, Bairoch A, Wu CH, Barker WC, Boeckmann B, Ferro S, Gasteiger E, Huang H, Lopez R, Magrane M, Martin MJ, Natale DA, ODonovan C, Redaschi N, Yeh LSL (2004). UniProt: the universal protein knowledgebase. Nucleic Acids Res..

[19] Kozomara A, Birgaoanu M, Griffiths-Jones S (2018). mirbase: from microrna sequences to function. Nucleic Acids Res..

[20] Köster J, Rahmann S (2018). Snakemake—a scalable bioinformatics workflow engine. Bioinformatics.

[21] Di Tommaso P, Chatzou M, Floden EW, Barja PP, Palumbo E, Notredame C (2017). Nextflow enables reproducible computational workflows. Nat Biotechnol.

