# Supplementary material for: FASTAFS: file system virtualisation of random access compressed FASTA files
Source: BMC Bioinformatics. 2021 Nov 1;22:535. doi: 10.1186/s12859-021-04455-3 (PMC8558547; doi:10.1186/s12859-021-04455-3)
Supplement: Supplementary file 1 — Additional file 1. Supplementary Figures. [file 12859_2021_4455_MOESM1_ESM.docx]

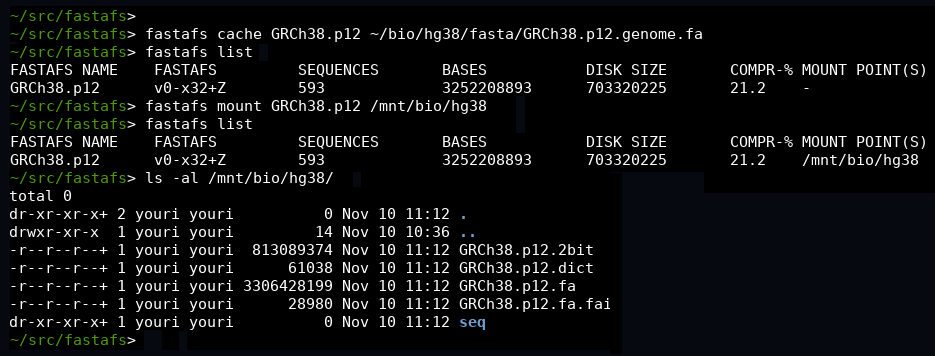


**Figure S1A: fastafs cache, mount & list**

Screenshot of several fastafs commands: it starts by creating an archive using fastafs cache, followed by requesting the archives present on the system with fastafs list. It then mounts the archive to a mount point using fastafs mount. When the archives present at the system are listed with fastafs list again, the active mount point is shown. When we perform a system directory listing (ls), the virtual files and sizes are shown.


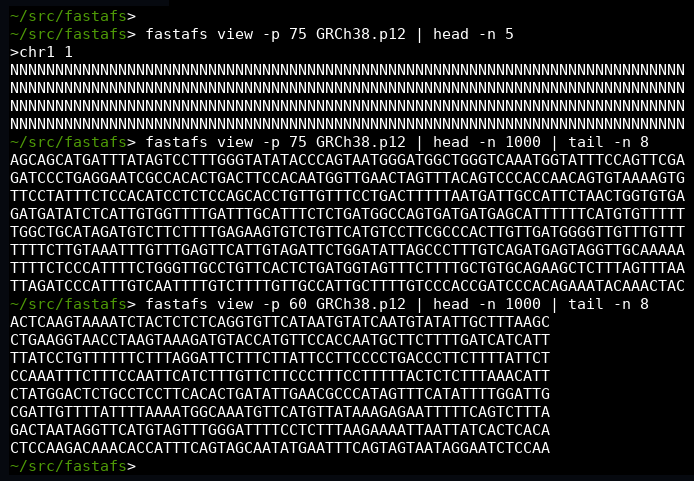


**Figure S1B: fastafs view**

The fastafs view command writes directly to *stdout*. The padding size can be controlled with the -p argument.


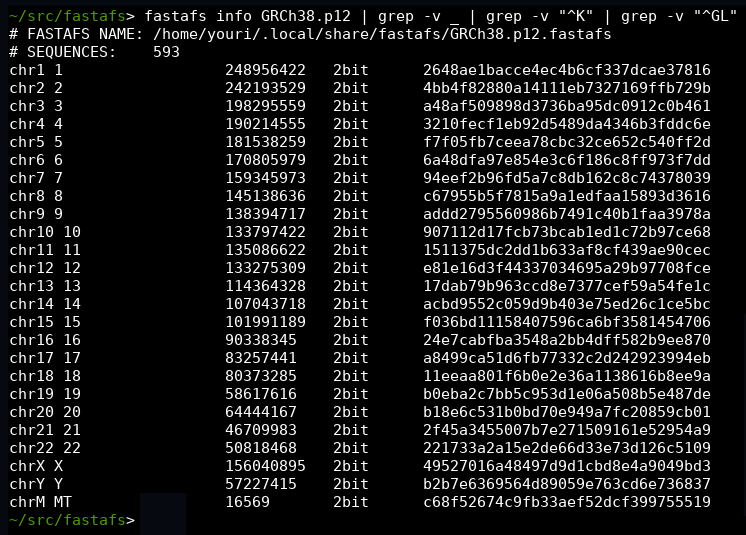


**Figure S1C: fastafs info**

The command fastafs info shows general and per-sequence information for a given archive. The ENA compatible md5 checksums are provided in the last column.


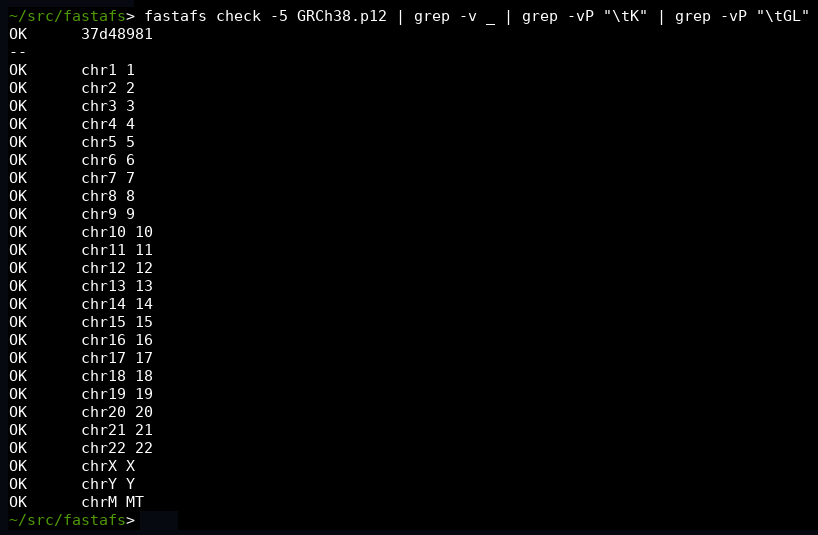


**Figure S1D: fastafs check**

The fastafs check command checks the file integrity using a crc32 checksum. Using the optional -5 argument, the per-sequence md5 checksum can be verified as well.


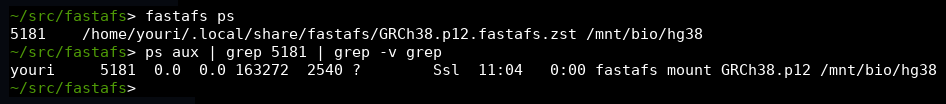


**Figure S1E: fastafs ps**

The fastafs ps command can be used to retrieve all running instances of FASTAFS with corresponding process id’s and mount points.


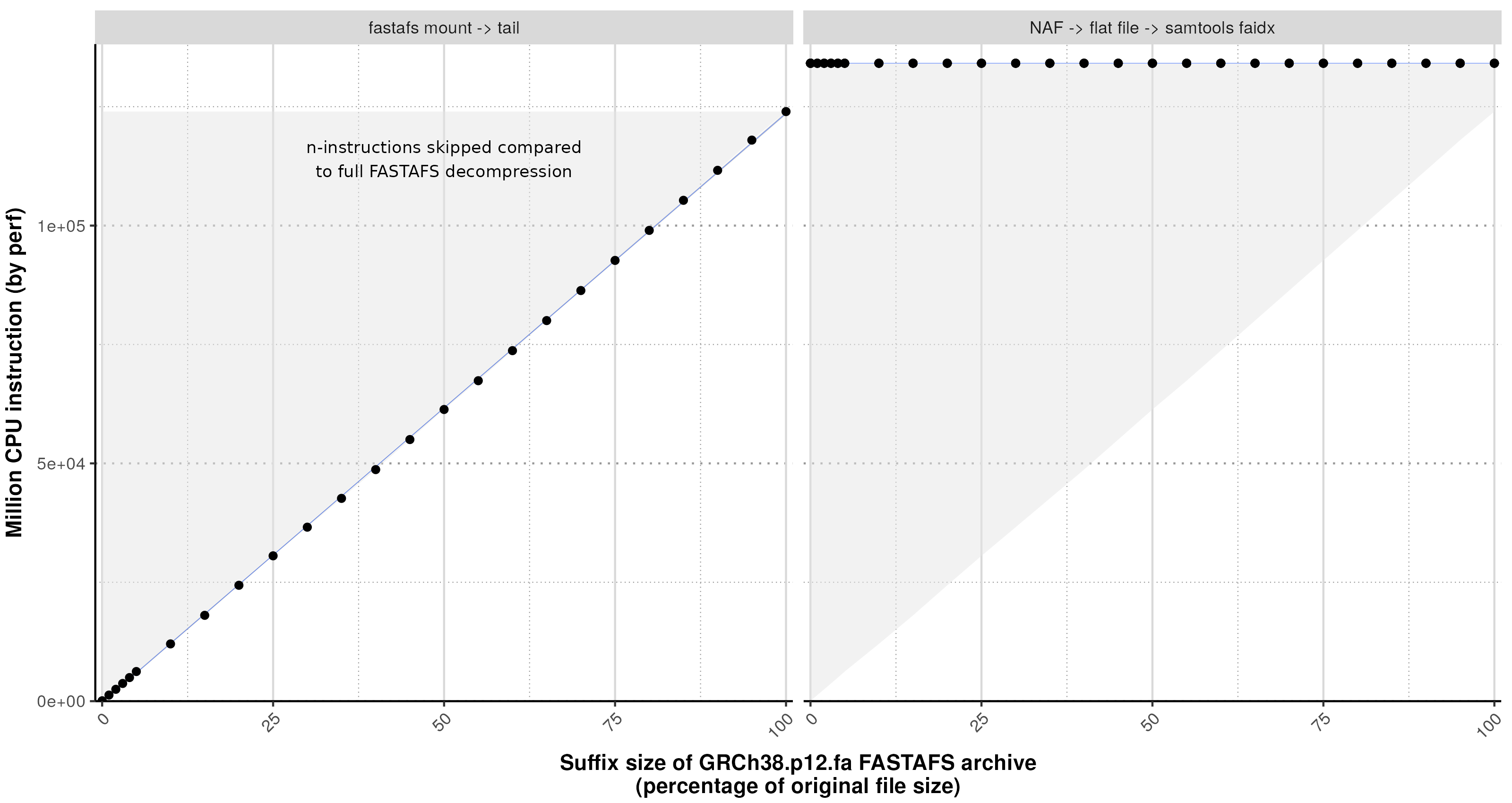


**Figure S2: CPU instructions to access FASTA and *fai*-index**

(**left**) The number of CPU instructions, determined by perf stat, the fastafs mount process needed when different sized suffixes of GRCh38.p12 were requested using tail -c. As the size of the suffix increases, the number of CPU instructions needed increases linearly. (**right**) The number of CPU instructions needed for to decompress a NAF archive using unnaf, followed by samtools faidx to generate the *fai*-index. The additional performance costs by the ext4 filesystem for accessing suffixes from the file on disk were not assessed because the mount process runs in kernel space rather than in user space.
